# Single-Cell Sequencing Reveals the Transcriptome and TCR Characteristics of pTregs and *in vitro* Expanded iTregs

**DOI:** 10.3389/fimmu.2021.619932

**Published:** 2021-03-31

**Authors:** Zhenzhen Hui, Jiali Zhang, Yu Zheng, Lili Yang, Wenwen Yu, Yang An, Feng Wei, Xiubao Ren

**Affiliations:** ^1^Department of Biotherapy, Tianjin Medical University Cancer Institute and Hospital, Tianjin, China; ^2^National Clinical Research Center for Cancer, Tianjin, China; ^3^Key Laboratory of Cancer Prevention and Therapy, Tianjin Medical University Cancer Institute and Hospital, Tianjin, China; ^4^Tianjin's Clinical Research Center for Cancer, Tianjin, China; ^5^Key Laboratory of Cancer Immunology and Biotherapy, Tianjin Medical University Cancer Institute and Hospital, Tianjin, China; ^6^Department of Immunology, Tianjin Medical University Cancer Institute and Hospital, Tianjin, China

**Keywords:** single-cell sequencing, *in vitro*, regulatory T cells, colorectal cancer, chemokine receptors

## Abstract

Regulatory T cells (Tregs) play a critical role in the maintenance of immune tolerance and tumor evasion. However, the relative low proportion of these cells in peripheral blood and tissues has hindered many studies. We sought to establish a rapamycin-based *in vitro* Treg expansion procedure in patients diagnosed with colorectal cancer and perform single-cell sequencing to explore the characteristics of Treg cells. CD25+ cells enriched from peripheral blood mononuclear cells (PBMC) of colorectal tumor patients were cultured in X-VIVO15 medium, supplemented with 5% human AB serum, L-glutamine, rapamycin, interleukin-2 (IL-2), and Dynabeads human Treg expander for 21 days to expand Tregs. Treg cells with satisfactory phenotype and function were successfully expanded from CD4+CD25+ cells in patients with colorectal cancer. The median expansion fold was 75 (range, 20–105-fold), and >90.0% of the harvest cells were CD4+CD25+CD127^dim/−^ cells. The ratio of CD4+CD25+Foxp3+ cells exceeded 60%. Functional assays showed that iTregs significantly inhibited CD8+T cell proliferation *in vitro*. Single-cell sequencing showed that the transcriptome of pTreg (CD4+CD25+CD127^dim/−^ cells isolated from PBMC of colorectal cancer patients) and iTreg (CD4+CD25+CD127^dim/−^ cells expanded *in vitro* according to the above regimen) cells were interlaced. pTregs exhibited enhanced suppressive function, whereas iTregs exhibited increased proliferative capacity. TCR repertoire analysis indicated minimal overlap between pTregs and iTregs. Pseudo-time trajectory analysis of Tregs revealed that pTregs were a continuum composed of three main branches: activated/effector, resting and proliferative Tregs. In contrast, *in vitro* expanded iTregs were a mixture of proliferating and activated/effector cells. The expression of trafficking receptors was also different in pTregs and iTregs. Various chemokine receptors were upregulated in pTregs. Activated effector pTregs overexpressed the chemokine receptor CCR10, which was not expressed in iTregs. The chemokine CCL28 was overexpressed in colorectal cancer and associated with poor prognosis. CCR10 interacted with CCL28 to mediate the recruitment of Treg into tumors and accelerated tumor progression. Depletion of CCR10+Treg cells from tumor microenvironment (TME) could be used as an effective treatment strategy for colorectal cancer patients. Our data distinguished the transcriptomic characteristics of different subsets of Treg cells and revealed the context-dependent functions of different populations of Treg cells, which was crucial to the development of alternative therapeutic strategies for Treg cells in autoimmune disease and cancer.

## Introduction

Regulatory T cells (Tregs) play a critical role in the regulation of self-tolerance and homeostasis (1–3). Tregs are classically defined as CD4+CD25+FoxP3+ cells, and they can *in vitro* and *in vivo* suppress CD4+ and CD8+ T cell-mediated immune responses through multiple mechanisms, including expression of CTLA-4, inhibitory cytokines secretion, metabolic disruption, granzyme-mediated cytotoxicity, and dendritic cell inhibition (4, 5). Many studies have demonstrated that CD4(+)CD25(+)CD127^dim/−^ was a surrogate phenotype to identify human Treg cells (6, 7). Models of graft-vs. host disease (GVHD) and autoimmune disease have evaluated the immunosuppressive properties of Treg cells (3, 8–10). Adoptive Treg cell transfer therapy was limited by the relative scarcity of natural Treg cells in human peripheral blood and the difficulty of *in vitro* amplification. Thus, Treg cell expansion and modification therapies that enhance suppressive function are of significant interest for the treatment of autoimmune diseases (11). Several studies have reported *in vitro* large-scale expansion of human Treg cells with a strong immunosuppressive ability for autoimmune diseases treatment (3, 12, 13).

Regulatory T cells also play a crucial role in modulating tumor immunosuppressive microenvironments. Intratumoral infiltration of Treg cells occurs in multiple human cancers (14–16). A series of studies have demonstrated that Treg cells in peripheral blood of tumor patients are significantly increased compared with normal volunteers, and levels are positively correlated with tumor stage (17, 18). Treg infiltration of cancer tissues is also significantly increased compared with adjacent tissues, and activation and functional signature genes, such as FoxP3, CTLA-4, and TNFRSF9, with a stronger immunosuppressive capacity are upregulated (19, 20). Moreover, the proportion of Treg in the tumor tissues of patients with colorectal cancer was higher than that in the normal mucosa, and also higher in regional lymph nodes nearest the tumor than distant parts of regional lymph nodes and non-regional lymph nodes (21). Tumor and stromal cells secreted chemokines that attract Treg migration to the tumor and promote immune escape (22, 23). Treg cells upregulate various chemokine receptors in an context-dependent manner, migrate to tumor microenvironment (TME) and inflammatory sites, and play an important role in inhibiting anti-tumor immune responses (24). The chemokine receptor CCR4 is expressed by effector Treg cells in gastric and lung cancer tissues (25–27). In addition to providing chemotactic navigation to guide Treg cells into tumors, the chemokine receptor CCR8 also supports the function and stability of Tregs, which is associated with poor prognosis of several cancers (28, 29). Therefore, targeting tumor-infiltrating Tregs chemokine receptors may be an attractive approach to elicit effective anti-tumor immune responses in patients.

However, despite the importance of Tregs in immune tolerance and tumor progression and their role as potential therapeutic targets, the *in vitro* expansion and transcriptome characteristics of Treg cells in tumor patients have not been established to date. Hence, we established an *in vitro* expansion protocol for Tregs from peripheral blood CD25+ cells of colorectal cancer patients (3, 12, 13, 30, 31), and then applied 10X Genomics single-cell transcriptome/TCR sequencing to compare the similarities and differences between peripheral blood Treg cells (CD4+CD25+CD127^dim/−^ cells isolated from PBMC of colorectal cancer patients, pTregs) and *in vitro* expanded Treg cells (CD4+CD25+CD127^dim/−^ cells expanded *in vitro* according to our culture regimen, iTregs) based on RNA expression and TCR clonality (6). Treg cells with satisfactory phenotype and function were successfully amplified from CD25+ cells in patients with colorectal tumors. Single-cell analysis showed that the transcriptomes of pTregs and iTregs were similar with an interlaced transcriptome but different TCR repertoires. Pseudotime development analysis identified three Treg differentiated states: activated/effector, resting, and proliferative Tregs. Different trafficking receptor expression profiles were also observed in pTreg and iTreg cells. Multiple chemokine receptors were upregulated in pTregs. The differential expression of chemokine receptors revealed the functional adaptability and tissue specificity of Treg cells. These data could aid in better understanding of the characteristics of Treg cells and pave the way for the identification of more specific and safer therapeutic targets in cancer therapy (32).

## Materials and Methods

### *In vitro* Expansion of iTregs From CD25+ Cells

**Figure 1:**
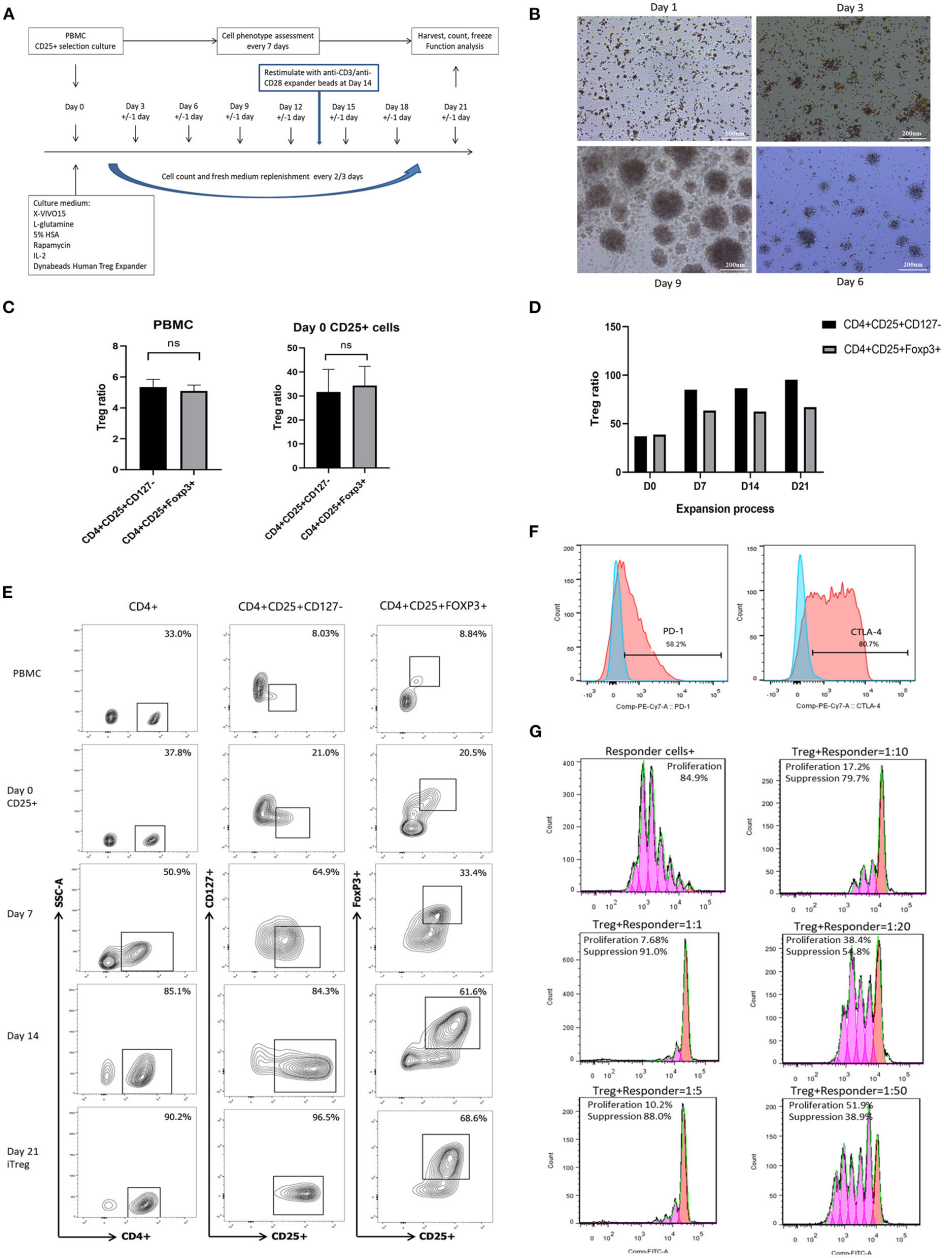
*In vitro* expansion, phenotype identification, and function assay of iTreg cells. **(A)** Flowchart of iTreg expansion process. CD25+ cells were expanded in the presence of Anti-CD3/anti-CD28 expander beads, IL-2, and rapamycin during 21 days. **(B)** Photograph captured under microscope during iTreg expansion; (C) The ratio of CD4+CD25+CD127^dim/−^ and CD4+CD25+FoxP3+ cells in PBMC and initial day 0 enriched CD25+ cells; **(D)** CD4+CD25+ CD127^dim/−^ and CD4+CD25+FoxP3+ ratio changes during 21 days expansion. **(E)** Flow cytometry results of CD4+CD25+ CD127^dim/−^ and CD4+CD25+FoxP3+ ratio in PBMC, Day 0 enriched CD25+ cells, day 7, day 14 and harvested iTreg cells at day 21. **(F)** PD-1 and CTLA-4 expression on pTreg and expanded iTreg cells; **(G)** CFSE-labeled suppression assay of expanded iTreg cells at a Treg to responder cell ratio of 1:1, 1:5, 1:10, 1:20, and 1:50.

With prior patient consent and under approval of the institutional review board, peripheral blood mononuclear cells (PBMC) were isolated from whole blood samples obtained from colorectal cancer patients using density gradient centrifugation. CD25+ cells were obtained by positive magnetic cell sorting using CD25+ cell isolation beads (CD25 MicroBeads II, human, Miltenyi Biotec, Bergisch Gladbach, Germany) according to the manufacturer's instructions. The CD25- “left-over” cell fraction was viably frozen in fetal bovine serum (FBS, Gibco, Grand Island, New York, USA) supplemented with 10% dimethyl sulfoxide and stored at −80°C until used. CD25+ cells were cultured in X-VIVO15 (Lonza, Brainel'Alleud, Belgium) supplemented with 2 mM L-glutamine (Lonza, Brainel'Alleud, Belgium), 0.1 mg/mL rapamycin (MACS GMP rapamycin, Miltenyi Biotec, Bergisch Gladbach, Germany), 500 U/mL IL-2 (recombinant human IL-2, PeproTech, Rocky Hill, NJ, USA), and 5% human AB serum at 37°C in 5% CO_2_. Anti-CD3/anti-CD28 expander beads (Dynabeads Human Treg Expander, Gibco, Grand Island, NY, USA) were added at a bead-to-cell ratio of 4:1 at day 0 and 1:1 at day 14 to the cell cultures. Cell counts and addition of fresh culture medium were conducted every 2 to 3 days (Figure 1A). Flow cytometry (FACS Canto II, BD Biosciences, San Jose, CA, USA) was used to determine cell phenotype every 7 days. At day 21, after removal of anti-CD3/anti-CD28 expander beads, iTreg cells were harvested, and functional analysis was performed.

### Assessment of iTreg Phenotype and Function

Cell phenotype was determined every 7 days during iTreg expansion and at harvest by staining with directly conjugated mouse anti-human antibodies (mAbs) against CD4 (Percp, clone: RPA-T4, Biolegend, San Diego, CA), CD25 (PE, clone: BC96, Biolegend, San Diego, CA), CD127 (FITC, clone: HIL-7R-M21, BD Biosciences, San Jose, CA), CD8 (PE/Cy7, clone: HIT8a, Biolegend, San Diego, CA), and CD19 (FITC, clone: HIB19, Biolegend, San Diego, CA). Cells were intracellularly stained with FoxP3 (AF647, clone: 259D/C7, BD Biosciences, San Jose, CA) using a FoxP3/Transcription Staining Buffer Set (eBioscience™ Foxp3/Transcription Factor Staining Buffer Set, Invitrogen, Carlsbad, CA) according to the manufacturer's instructions.

To determine the ability of iTregs to inhibit the proliferation of effector T cells *in vitro*, cryopreserved autologous CD25- cells were thawed, washed, and labeled with 5 mM carboxyfluorescein succinimidyl ester (CFSE) (CellTrace™ CFSE Cell Proliferation Kit, Invitrogen, Eugene, OR) (3, 13). Next, CFSE-labeled CD25- cells (hereafter referred to as responder cells) were coincubated with expanded iTreg cells at Treg-to-responder ratios of 1:1, 1:5, 1:10, 1:20 and 1:50. Co-cultures were routinely performed in triplicate in U-bottom 96-well-plates. In the majority of experiments, a total of 1 × 10^5^ Tregs (1:1 ratio), 2 × 10^4^ Tregs (1:5 ratio), 1 × 10^4^ Tregs (1:10 ratio), 5 × 10^3^ (1:20 ratio), and 2 × 10^3^ (1:50 ratio) were added to a total of 1 × 10^5^ responder cells. Co-cultures were stimulated with anti-CD3/anti-CD28 expander beads at a bead-to-cell ratio of 1:1. Co-cultures were harvested after 4 days, stained with dead cell dye NIR (Zombie NIR^TM^ Dye, Biolegend, San Diego, CA); CD3 (APC, clone: HIT3a, Biolegend, San Diego, CA), CD4, and CD8 mAbs; and proliferation as measured by CFSE dilution was analyzed by flow cytometry. Negative controls included responder cells alone and Treg + responder cells at a ratio of 1:1 without the addition of anti-CD3/anti-CD28 expander beads. The positive control included responder cells alone with the addition of antiCD3/anti-CD28 expander beads (13). The percentage of suppression = {1-(%proliferation in the presence of Tregs/%proliferation in the absence of Tregs)} × 100% (33). The suppression rate was ≥80.0% for dividing cells at a Treg to responder cell ratio of 1:1, ≥60.0% for dividing cells at a Treg to responder cell ratio of 1:5, and ≥50.0% suppression for dividing cells at a Treg to responder cell ratio of 1:10.

### Single-Cell Transcriptome/TCR Sequencing of Tregs

Single-cell transcriptome/TCR sequencing of pTregs (CD4+CD25+CD127^dim/−^ cells isolated from PBMC of colorectal cancer patients) and iTregs (CD4+CD25+CD127^dim/−^ cells expanded *in vitro* according to our culture regimen) was performed to compare the similarities and differences in RNA expression and TCR repertoires. With informed consent, 100 ml heparinized blood was obtained from a 62-year-old female patient diagnosed with colon cancer. PBMCs were isolated by ficoll density separation. Half of the fresh PBMCs were used for sorting CD4+CD25+CD127^dim/−^ pTreg cells (CD4+CD25+CD127^dim/−^ Regulatory T Cell Isolation Kit II, human, Miltenyi Biotec, Bergisch Gladbach, Germany) for single-cell sequencing, and the other half were sorted for CD25+ cells for iTreg expansion. After 21 days of expansion, CD4+CD25+CD127^dim/−^ iTreg cells were isolated from the culture system using the same regulatory T Cell isolation Kit for single-cell sequencing.

The CD4+CD25+CD127^dim/−^ Treg cells were resuspended in DPBS (DPBS, no calcium, no magnesium, Gibco, Grand Island, NY, USA) and adjusted to 700–1,200 cells/μl. Standard protocol of the chromium single cell 3′ kit was performed to load cells to capture 3,000 cells/chip position (V2 chemistry). Library construction and all the remaining procedures were carried out according to the standard manufacturer's protocol.

### Single-Cell Bioinformatics and Statistical Analysis

Cell Ranger pipelines (v2.1.1, 10X Genomics) was used to process single-cell sequencing data to obtain T-cell clonotypes and RNA expression profiles associated with individual single-cell barcodes. Unique paired TRαTRβ V-gene/CDR3/J-gene sequences were used to determine clonotypes. In order to prevent the artificial inflation of clone counts due to the reduction of gene sequence information, only cells with one α-chain and one β-chain were assigned clonotypes. Low-quality single cells with detectable genes fewer than 200 were excluded. Subsequent analysis also removed the genes <10 read counts and mitochondrial genes. Non-coding RNA genes were not investigated in our study.

All uniquely aligned reads with the same unique molecular identifiers (UMI), cell barcode, and gene annotation in the HDF5 format were merged to construct the gene expression matrix for the datasets. Convolution methods were used to normalize the raw single cell data. Randomized principal component analysis was used to decompose the normalized data. T-distributed Stochastic Neighbor Embedding (t-SNE) and Uniform Manifold Approximation and Projection (UMAP) projections was generated by the principal components, which were implemented with the R package Seurat (v2.3.0) (https://github.com/satijalab/seura). The edgeR package was used to compare the differential expression of selected genes (false discovery rate, FDR <0.05).

### Marker Gene Identification, Functional Enrichment, and Pseudotime Trajectory Analysis

The FindAllMarkers function in the Seurat package was used to identify the cluster-specific marker genes using normalized gene expression data, “find.markers” function was used to identify differentially expressed genes between two clusters. Metascape (http://metascape.org) and DAVID (https://david.ncifcrf.gov/home.jsp) were used to perform biological process enrichment analysis with significant differentially expressed genes in each cluster or subgroup. Pseudotime cell developmental trajectory analysis was analyzed by Monocle 2 package (v2.8.0) to explore the cell-state transitions.

### Multiplex Immunohistochemistry Staining and Statistics

Formalin fixed paraffin-embedded specimens from 70 patients diagnosed with colorectal cancer who underwent surgical resection of tumor tissue were included in this study and analyzed by multiplex fluorescent immunohistochemistry (IHC) under an approved Institutional Review Board protocol. These patients did not receive chemoradiotherapy or immunotherapy before surgery. The primary antibodies and IHC metrics were: rabbit anti-human CCL28 (Invitrogen, 1:1500), rabbit anti-human CCR10 (Proteintech, 1:800), mouse anti-human FoxP3 (Abcam, 1:500). Multiplex fluorescent staining was obtained using Opal 4-Color Manual IHC Kit (NEL810001KT, PerkinElmer). Slides were scanned and visualized using the TissueFAXSi-plus imaging system (TissueGnostics, Vienna, Austria; acquisition software: TissueFAXS v7.0.6245) equipped with a digital Pixelink color camera (PCO AG). Multispectral images were analyzed with StrataQuest software v7.0.1.165 (TissueGnostics). The correlations of different biomarkers were evaluated by Spearman's correlation analysis (*P* < 0.05). Kaplan–Meier Log Rank test was used to perform univariate survival analysis. SPSS (version 25.0, IBM) and GraphPad Prism (version 8.0.1, US) were used for statistical analysis.

## Results

### *In vitro* iTreg Expansion, Phenotype Identification, and Functional Suppression Assay

A total of 11 culture procedures were performed. Clinicopathological data of the included patients are provided in Table 1. The initial CD25+ cells contained a median of 7.1 × 10^6^ (range, 0.82 × 10^6^ to 25 × 10^6^), and the expansion achieved 1.48 × 10^8^ (range, 1.6 × 10^7^ to 2.2 × 10^8^) after 21 days of culture with a median amplification of 75-fold (range, 20–105-fold). The CD25+ cells clumped together on days 2–3 after culture and then proliferated dramatically (Figure 1B). The ratio of CD4+CD25+CD127^dim/−^ and CD4+CD25+FoxP3+ cells was 5.34% (range, 3.85–6.86%) and 5.08% (range, 3.73–6.50%) in PBMCs, respectively, and 31.7% (range, 19.6–41.5%) and 34.3% (range, 22.6–42.6%) in day 0 enriched CD25+ cells, respectively (Figure 1C). During expansion, the proportion of CD4+CD25+CD127^dim/−^ cells increased gradually and was >90.0% on day 21. CD4+CD25+Foxp3+ cells accounted for >60% of the harvested cells on day 21 (Figure 1D), meeting the proposed criteria based on previous studies (34). The proportion of CD4+CD25+CD127^dim/−^ and CD4+CD25+FoxP3+ in PBMCs, day 0 enriched CD25+ cells, day 7, day 14, and day 21 harvested iTreg cells measured by flow cytometry were presented in Figure 1E. Moreover, compared to pTregs, *in vitro* expanded iTregs highly expressed the immune checkpoint molecules PD-1 and CTLA-4 (Figure 1F). The immune suppressive capacity of expanded iTregs (on day 21) was assessed in a CFSE-based coincubation assay. CD25- cells were thawed and incubated with the expanded iTreg cells at Treg to responder ratios of 1:1, 1:5, 1:10, 1:20, and 1:50 in triplicate in U-bottom 96-well-plates. Suppressive functional assays showed that iTreg cells significantly inhibited CD8+T cell proliferation *in vitro*. The rate of suppression of dividing cells was 82.5–91.0% at a Treg to responder cell ratio of 1:1, 65.2–88.0% at a Treg to responder cell ratio of 1:5, 54.1–79.7% at a Treg to responder cell ratio of 1:10, and 40.3–54.8% at a Treg to responder cell ratio of 1:20. There was also a slight inhibition of CD8+T cell proliferation at a Treg to responder cell ratio of 1:50 (Figure 1G).

**Table 1:** Clinicopathological features of the included patients for iTreg expansion.

**Patients**	**Sex**	**Age**	**Tumor location**	**Pathologic types**	**MMR status**	**TNM stage**	**Expansion (Fold)**
1	Male	51	Rectum	Adeno	pMMR	IIa	74
2	Male	50	Rectum	Adeno	pMMR	IIIb	46
3	Male	36	Right colon	Adeno	pMMR	IIIb	105
4	Female	70	Left colon	Adeno	pMMR	IVc	20
5	Female	51	Left colon	Adeno	pMMR	IVa	53
6	Female	41	Rectum	Adeno	pMMR	IIIc	75
7	Male	55	Left colon	Adeno	dMMR	IIb	89
8	Female	63	Left colon	Adeno	dMMR	IVa	58
9	Male	67	Sigmoid	Adeno	pMMR	IIa	94
10	Male	74	Right colon	Adeno	dMMR	IIIb	87
11^*^	Female	61	Sigmoid	Adeno	pMMR	IIIb	93

* *Patient who donated blood for single-cell sequencing analysis; dMMR, mismatch repair deficiency; pMMR, proficient mismatch repair; TNM, Tumor-Lymph Node-Metastasis*.

### Overall Single-Cell Transcriptome Characteristics of pTreg and iTreg Cells

**Figure 2:**
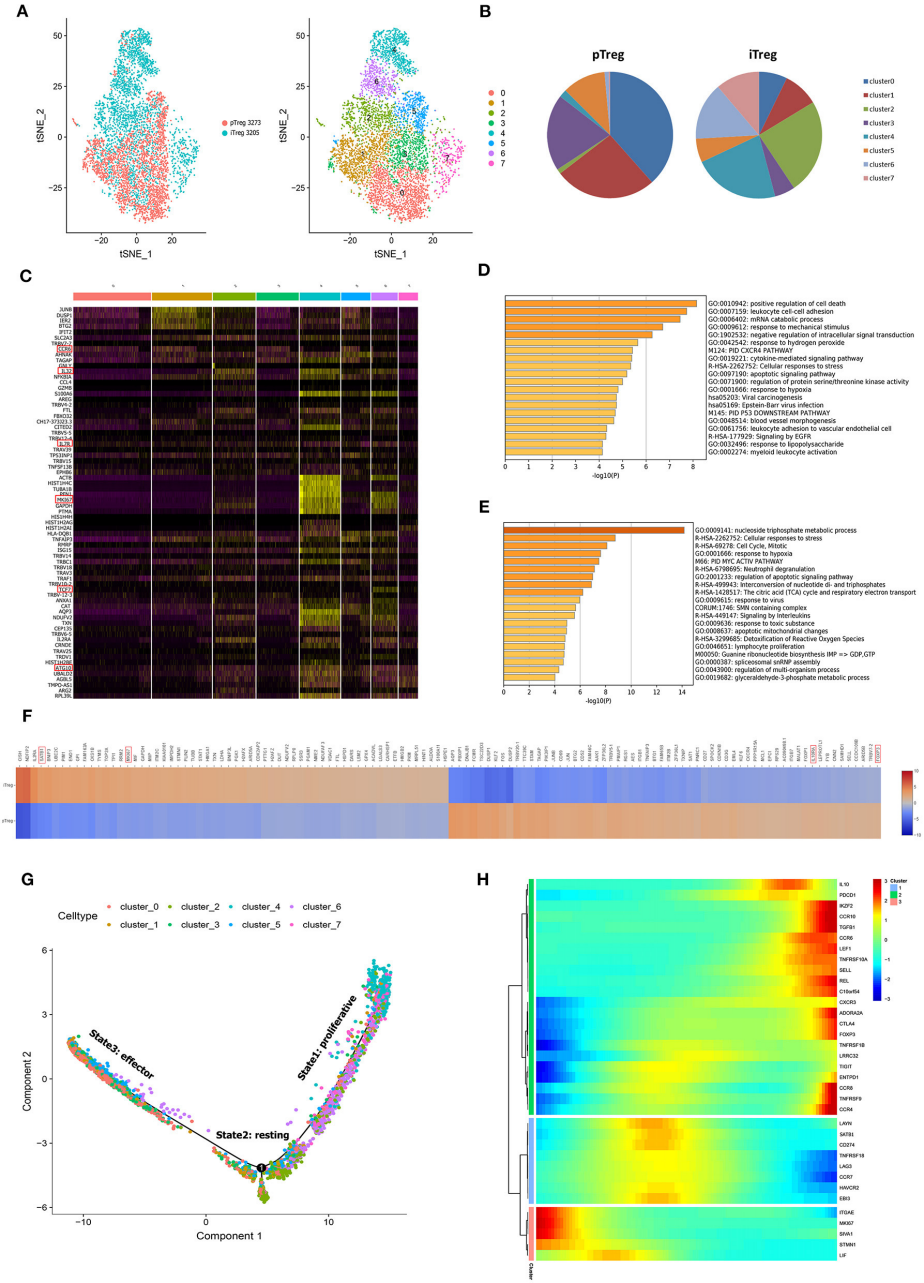
Overall single-cell transcriptome characteristics of pTreg and iTreg cells. **(A)** The t-SNE projection of 3,273 pTreg and 3,205 iTreg cells, showing the formation of 8 main clusters. **(B)** PTreg and iTreg cells distribution in each clusters. **(C)** Heatmap showed the top 10 differential expressed genes of 8 clusters. **(D,E)** Functional enrichment analysis of pTreg and iTreg cells by metascape (Barplot). **(F)** Comparison of significantly differential expressed genes between pTreg and iTreg cells; **(G)** Pseudo-time development trajectories of Treg cells; **(H)** Changes of 35 Treg signature genes expression in pseudo-time trajectory.

To study the gene expression profiles of Treg cells, pTregs and iTregs samples from the same patient were processed using a 10X Genomics single-cell instrument for single-cell RNA extraction and ctDNA library construction followed by next-generation sequencing (35). After initial quality filtering, we acquired a single-cell transcriptome from a total of 3,273 pTreg cells and 3,205 iTreg cells. We first used tSNE for dimensionality reduction to capture the overall similarity and differences between pTregs and expanded iTregs. The transcriptome profiles of pTreg and iTreg cells were interlaced and divided into 8 clusters (Figure 2A). Because our iTreg cells were amplified *in vitro*, their culture conditions could not fully simulate the cell microenvironment *in vivo*, so the cluster compositions of pTreg and iTreg cells exhibited some discrepancies. Most pTreg cells were clustered in C0, C1, C3, and C5 clusters, whereas iTreg cells were relatively uniform, with more cells distributed in clusters C2, C4, C6, and C7 clusters (Figure 2B). Cluster differential expression gene analysis showed that memory marker CCR6 was overexpressed by C0, C1, C3, and C5 (36), survival marker IL7R was slightly up-regulated in C3 (37), and C5 characteristically expressed thymic-derived marker Aire (autoimmune regulator). Aire encodes a transcriptional regulator that drives the ectopic expression of peripheral tissue-specific antigens by medullary thymic epithelial cells, and was critical for maintaining immune tolerance and tissue/organ specificity of Treg cells (38–40). Whereas, C2 overexpressed IL32, C4 highly expressed Mki67, and showed strong proliferative ability, C6 was relatively naive and overexpressed TCF7, and autophagy gene ATG10 was upregulated in C7 (Figure 2C). Thus, pTregs have more memory function and tissue/organ specificity, and iTregs have stronger proliferative ability and inhibitory function. Comparison of differential expression genes between pTreg and iTreg cells identified that IL10RA and FOXP3 were slightly increased in pTregs, whereas Treg-differentiated transcription factors SATB1 and the proliferative index MKI67 were highly expressed in iTregs (Figure 2F). Functional enrichment showed that genes expressed at higher levels in pTregs were mainly enriched in the positive regulation of cell death and leukocyte cell-cell adhesion and negative regulation of intracellular signal transduction (Figure 2D), whereas genes upregulated in iTregs were more focus on nucleoside triphosphate metabolic processes, cell cycle, mitosis, and regulation of apoptotic signaling pathways (Figure 2E). Next, we compared the differences in the metabolic pathways between pTregs and iTregs, found that iTregs upregulated in glyceraldehyde-3-phosphate metabolic process and fructose and mannose metabolism, whereas iTregs mainly involved in the regulation of protein and phospholipid metabolic process.

To better understand cell relationship between clusters, we used Monocle 2 to construct the pseudotime cell developmental trajectories of 8 clusters based on expression data. To further address the trajectories, we defined scores of proliferation, resting, and activation based on previously defined 35 Treg signature genes, such as Foxp3, CTLA4, Helios, CCR8, LAYN, REL, IL10, TGFB1, ADORA2A, TNFRSF9, TNFRSF18, ITGAE, SELL, SATB1, CCR7, STMN1, and MKI67 (19, 28, 40–48). Figure 2H presents the expression changes in Treg signature genes based on pseudo-time trajectory analysis. Analyzing the pseudotime cell developmental trajectory in the context of these Treg functional genes, we found that State 1 had a strong proliferation capacity with high expression of STMN1 and MKI67. State 3 was more activated and suppressive, and Treg functional signature genes TNFRSF9, FoxP3, CTLA4, CCR8, ADORA2A, REL, TGFB1, and Helios were highly expressed in State 3. State 2 was a group of resting cells with high expression of CCR7 and lower expression of TNFRSF9 (11, 19) (Figures 2G,H). Thus, our heterogeneity analysis of Treg demonstrated that Tregs were a continuum composed of three main states.

### Single-Cell Transcriptome Analysis Identifies Three Differentiated Branches Within pTregs

**Figure 3:**
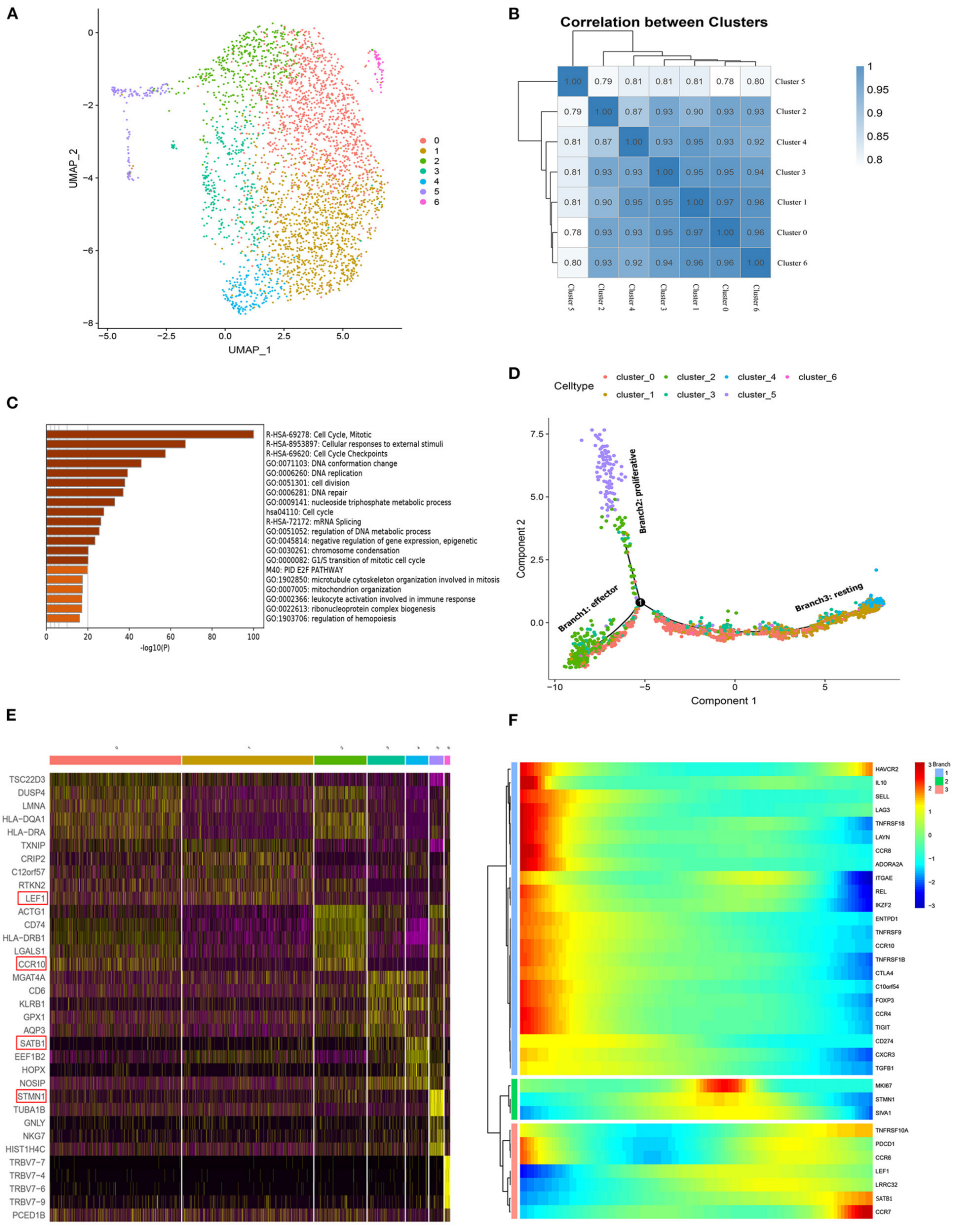
Single-cell transcriprome characteristics of pTreg cells. **(A)** The UMAP distribution of 3,273 pTreg cells, showing 7 main clusters; **(B)** Pearson correlation coefficient analysis between each clusters; **(C)** Function enrichment analysis of Cluster 5 by metascape (Barplot); **(D)** Pseudo-time trajectory analysis of pTreg by Monocle 2, Treg cells mainly divided into three branches: activated, resting and prolifertative Treg; **(E)** Heatmap showed the top 5 marker genes of 7 clusters. **(F)** The expression changes of 35 Treg signature genes in pseudo-time trajectory.

To gain insight into the intrinsic Treg cell heterogeneity in PBMCs, we applied unsupervised clustering in pTreg cells based on UMAP and identified 7 clusters (Figure 3A). The top 5 cluster marker genes are shown in Figure 3E. C0 upregulated the expression of chemokine receptor CCR10, C1 was naïve Treg characteristically expressed LEF1, C2 was more suppressive and overexpresssed the Treg functional gene such as CCR10, FoxP3, and CTLA-4, C3 highly expressed T cell activation marker CD6, C4 upregulated Treg differentiated transcription factor SATB1, C5 was more proliferative with high expression of MKI67, and C6 highly expressed TRBV7 genes. Pearson correlation coefficient analysis between each cluster revealed that C5-MKI67 exhibited reduced correlation with other clusters (Figure 3B). Function enrichment analysis showed that C5 mainly involved in cell cycle, mitosis, cellular responses to external stimuli, cell cycle checkpoints, DNA conformation change, and DNA replication (Figure 3C). Next, we used Monocle 2 for pseudo-time trajectory analysis and three main branches were observed within pTreg cells (Figure 3D). Cells in Branch 2 seemed to exhibit a proliferative phenotype, which was represented by C5 with high expres2sion of the proliferative genes TYMS, STMN1, and MKI67. In addition, a general gradient that separated resting and activated/effector Tregs was noted based on the transcriptional frequency of the typical effector and resting Treg genes TNFRSF9 and CCR7. Branch 1 included a group of activated/effector Treg cells with high expression of Treg activated and functional signature genes, such as TNFRSF9, CD103, AADORA2A, FOXP3, CTLA4, IL10, TGFB1, CCR8, and Helios. In contrast, cells in Branch 3 seemed to be in a resting state, and LEF1, CCR7, and Treg differentiated transcription factor SATB1 were highly expressed to maintain this state (Figures 3E,F). However, the Treg state was dynamic, and this phenotypic analysis could only indicate different differentiation states of cells at a certain time. Resting Tregs may represent a precursor population of Tregs that would convert into a fully functional Tregs upon activation.

### *In vitro* Expanded iTregs Are a Mixture of Proliferating and Effector Cells

**Figure 4:**
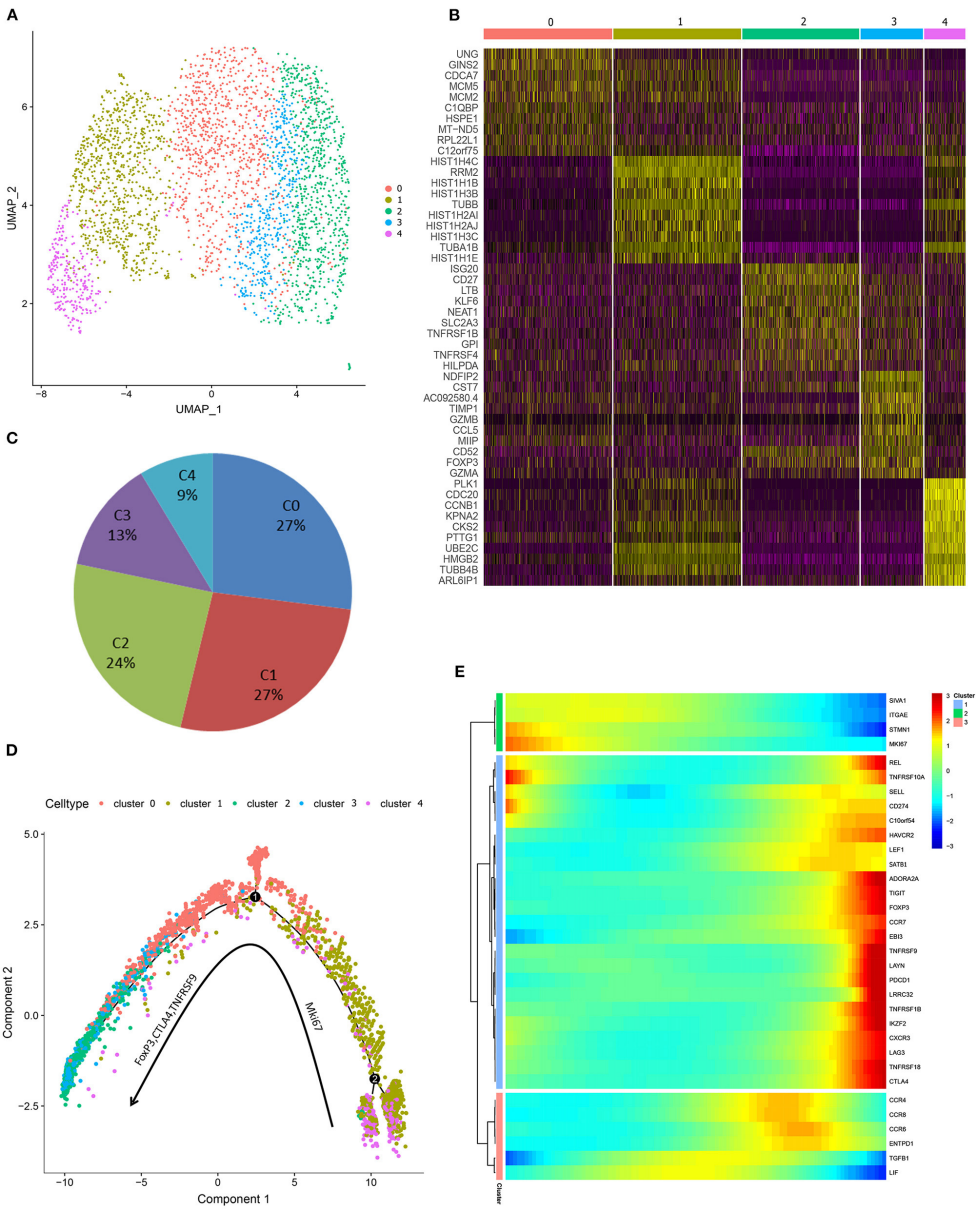
Single-cell transcriprome characteristics of the harvested iTreg cells. **(A)** The UMAP distribution of 3,205 iTreg cells, identifying 5 main clusters. **(B)** The top 10 marker genes of clusters. **(C)** Proportion of cells in each cluster. **(D)** Pseudo-time development trajectories of iTreg cells identified that *in vitro* expansion of iTreg was a dynamic and continuous process. **(E)** The expression changes of 35 Treg signature genes in pseudo-time trajectory.

To understand functional states of *in vitro* expanded iTregs, principal component analysis was used to generate UMAP projection, and 5 clusters were identified within iTreg cells (Figure 4A). The cell ratio and top 10 marker genes of each cluster are shown in Figures 4B,C. Functional enrichment of differential expression genes of C1-UNG was noted for double-strand break repair via break-induced replication and pyrimidine nucleotide metabolic processes. C1-HIST1H4C and C4-CDC20 exhibited high proliferative ability and functional enrichment mainly in cell cycle and mitosis. C2-CD27 was enriched in lymphocyte activation and leukocyte differentiation, and C3-FoxP3 upregulated Treg signature genes, resembling activated/effector Treg cells. In addition, its function was mainly focused on the positive regulation of cell death. Then, we applied pseudotime development trajectory analysis using Monocle 2 to further explore the development characteristics of iTreg cells and found that *in vitro* expansion of iTregs was a dynamic and continuous process (Figures 4D,E). The trajectory arose from proliferative clusters C4 and C1 and gradually developed into functional effector Treg cells (C0, C2, and C3) during expansion, with high expression of multiple functional signature genes of Tregs, such as FoxP3, CTLA-4, TNFRSF9, REL, ADORA2A, LAYN, and Helios. Therefore, the harvested iTregs included a mixture of proliferating and effector Treg cells, which were possibly transformed from CD4+CD25+ T cells.

### Distinct TCR Repertoires of pTreg and iTreg Cells

**Figure 5:**
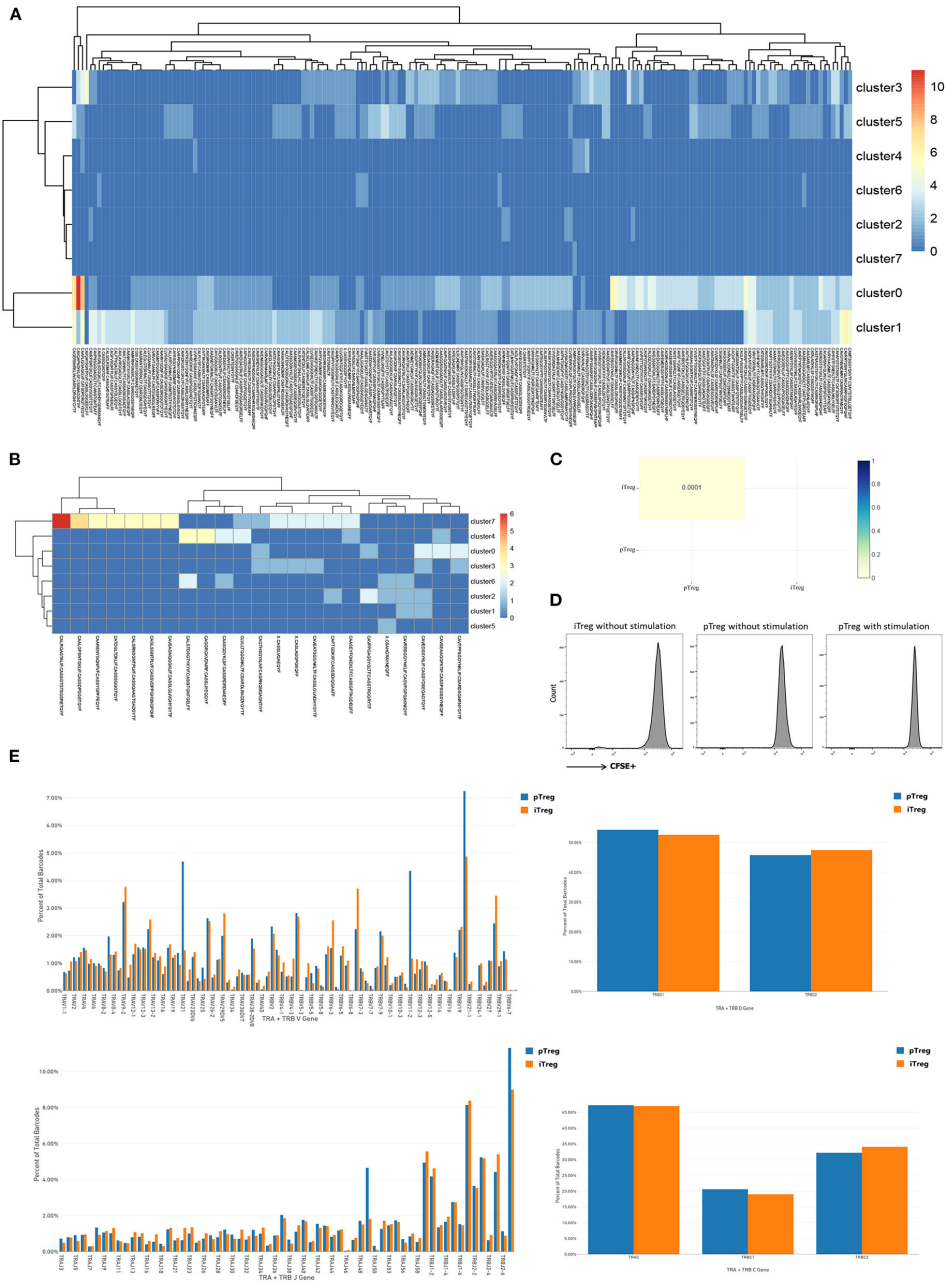
Distinct TCR repertoires of pTreg and iTreg cells. **(A)** TCR clones supported by more than two cells in each cluster in pTreg; **(B)** TCR clones supported by more than two cells in each cluster in iTreg; **(C)** TCR similarity comparison between pTreg and iTreg by Morisita-Horn; **(D)** CFSE-labeled proliferation of iTreg and pTreg cells without Anti-CD3/anti-CD28 expander beads, IL-2, and rapamycin stimulation, and CFSE-labeled proliferation of pTreg with Anti-CD3/anti-CD28 expander beads, IL-2, and rapamycin stimulation; **(E)** TCR VDJC genes usage analysis of pTreg and iTreg cells.

The observation that pTreg and iTreg cells were functionally similar and transcriptionally overlapped led us to ask whether iTregs originate from direct expansions of pre-existing proliferating Treg cells in peripheral blood. To determine whether the two are distinct populations and whether pTregs are precursors of iTregs, we first analyzed the TCR repertoire of the two cell types using VDJ tools software (49). These analyses identified low TCR repertoire overlap between iTreg and pTreg cells. TCR similarity measured by morisita-horn was 0.0001, and only 3 shared clonotypes and 18 shared CDR3s were noted (Figure 5C). Then, we tried to amplify pure pTreg cells according to the iTregs expansion protocol, but no obvious CFSE-labled proliferation was observed. CFSE-labeled proliferation of iTregs and pTregs without the addition of rapamycin, IL-2, and anti-CD3/anti-CD28 expander beads in the medium also showed no proliferation (Figure 5D). Thus, it was unlikely that pTregs are the primary origin of iTregs. We hypothesized that iTregs amplified *in vitro* were mainly transformed from activated CD4+CD25+ cells under the action of rapamycin, IL-2, and anti-CD3/anti-CD28 Dynabeads in the culture system. TCR abundance analysis of pTregs and iTregs showed that both populations were very diverse and polyclonal, and pTreg cells contained more large clones. There were 187 TCR clones with three or more cells in pTregs and 23 clones in iTregs. Heat maps show TCR clones supported by more than two cells in each cluster (Figures 5A,B). Analysis of VDJC gene usage revealed that certain VJ sequences were used more frequently in pTreg cells, such as TRAV21, TRBV11-2, TRBV19, TRAJ49, and TRBJ2-7. DC gene usage did not differ between the populations (Figure 5E). Thus, by comparing the TCR repertoires, we found that the TCR clonotypes of pTregs and iTregs were quite distinct, which further supported our hypothesis that iTregs were mainly transformed from conventional CD4+CD25+ T cells rather than pTreg cells.

### Different Trafficking Receptor Expression Profiles in pTregs and iTregs

**Figure 6:**
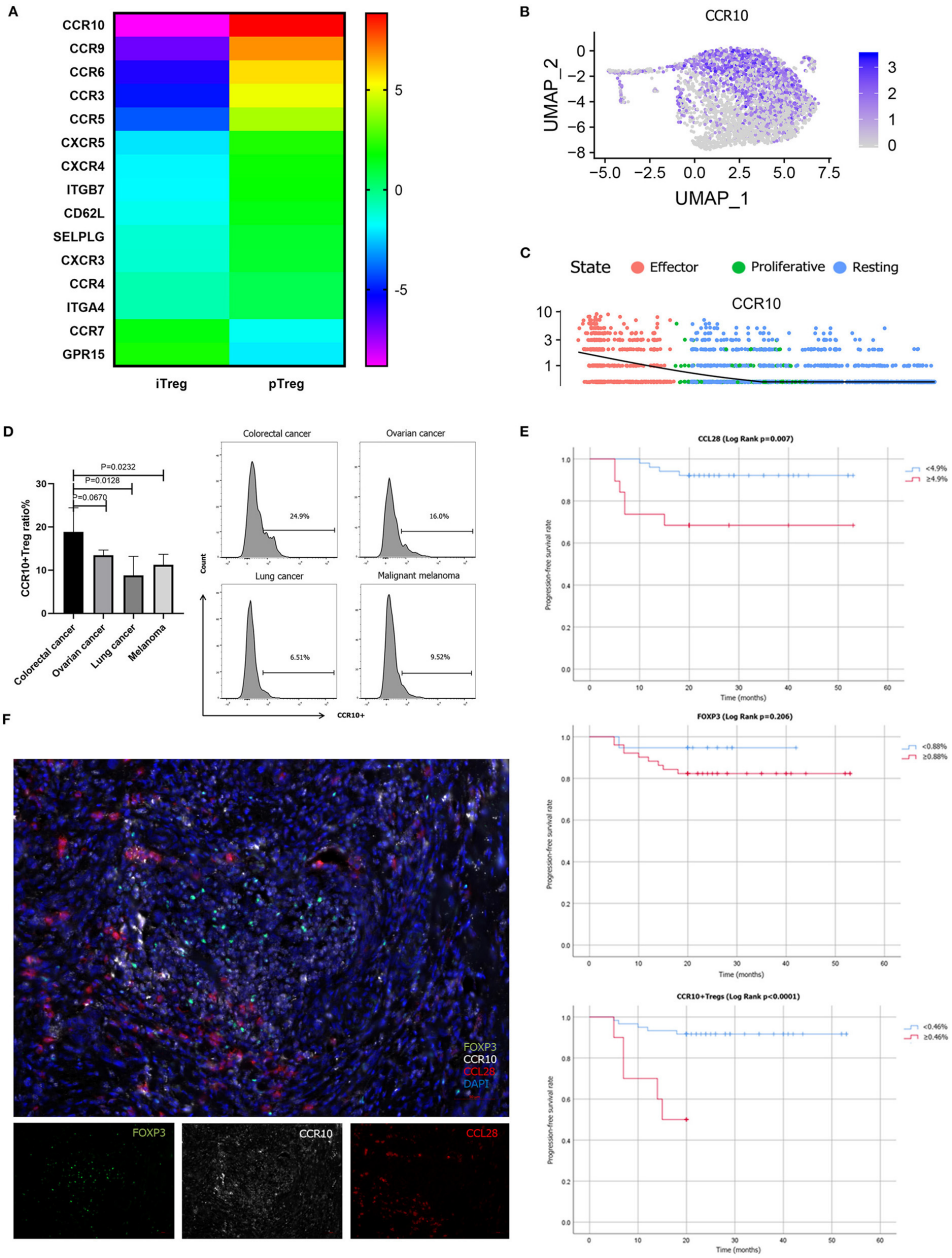
Different trafficking receptor expression profiles in pTregs and iTregs. **(A)** Comparison of different trafficking receptors between pTregs and iTregs; **(B)** Heatmap expression of CCR10 in pTreg cells; **(C)** CCR10 expression in the three differentiated states of pTreg cells; **(D)** Comparison of CCR10+Tregs ratio in peripheral blood between colorectal cancer and ovarian cancer, lung cancer and malignant melanoma; **(E)** Kaplan–Meier univariate survival analysis of CCL28, FoxP3, CCR10+Treg, and PFS; **(F)** Multiplex fluorescent IHC showed the physical juxtaposition of CCL28+, CCR10+, and FoxP3+ cells (20X).

The cluster composition between pTreg and iTreg cells showed some discrepancies, and further analysis showed that certain chemokine receptors were differentially expressed. To confirm this notion, we compared the various trafficking molecules involved in T cell transport, such as chemokine receptors (CKRs), integrins and selectins (50), and found that multiple chemokine receptors were highly upregulated in pTreg cells, including CCR10, gut homing receptor CCR9, memory T cell receptor CCR6, CCR3, and CCR5, indicating that pTreg cells exhibited increased organ or tissue specificity (Figure 6A). Interestingly, we noted that the chemokine receptor CCR10 was particularly highly expressed in activated effector pTreg cells, mainly in the pTreg C0 and C2 clusters, but almost absent in the expanded iTregs (Figures 6B,C). We further evaluated the proportion of CCR10+Treg in peripheral blood of colorectal cancer and compared with tumors from other sites. Flow cytometry analysis showed that, at the protein level, CCR10 expression was a bit higher than ovarian cancer in peripheral blood Treg of colorectal patients (Unpaired *t*-test, *P* = 0.0670), but much higher compared with lung cancer and malignant melanoma patients (Unpaired *t*-test, *P* = 0.0128; *P* = 0.0232; Figure 6D). The chemokine CCL28, which is a ligand of CCR10, is expressed in hypoxic areas of ovarian and liver tumors in an HIF-1α-dependent manner, and CCL28 recruits CCR10-expressing effector Tregs into the neoplasm to promote tumor growth (22, 23, 51). The expression of CCL28 was significantly positively correlated with the expression of HIF-1a in colorectal cancer in TCGA database (*R* = 0.15, *P* = 2.5e-05). Several studies have also confirmed that CCL28 was overexpressed in colorectal cancer tissues and associated with poor prognosis (52–54). To validate the interaction of CCR10 and CCL28 in colorectal tumor microenvironment at the protein level, we performed multiplex fluorescent IHC in tumor tissues from 70 colorectal cancer patients. Multiplex fluorescent staining showed the physical juxtaposition of CCL28+, CCR10+, and FoxP3+cells (Figure 6F). Pearson correlation analysis showed that the expression of CCL28 was significant correlated with CCR10, FoxP3 and CCR10+Treg infiltration in colorectal cancer tissues (*R* = 0.675, *P* < 0.0001; *R* = 0.474, *P* < 0.0001; *R* = 0.515, *P* < 0.0001). We used Kaplan-Meier univariate survival analysis to analyze the prognostic value of CCL28, FoxP3, and CCR10+Treg cells, and identified that both CCL28 and CCR10+Treg were poor predictors of progression-free survival (PFS) in colorectal patients (49.9 vs. 38.6 months, Log Rank *P* = 0.007; 49.4 vs. 14.8 months, Log Rank *P* < 0.0001), while FoxP3 was not an independent prognostic factor for PFS (40.1 vs. 45.5 months, Log Rank *P* = 0.206; Figure 6E). CCL28 secreted in tumor microenvironment enabled the recruitment and retention of effector CCR10+pTregs in colorectal cancer tissues through CCR10-CCL28 interaction to promote tumor progression. Depletion of CCR10+Treg cells from tumor tissues could be used as an effective treatment strategy for colorectal cancer patients. Thus, from this perspective, the relative lower trafficking receptor expression of iTreg cells and loss of CCR10 expression in expanded iTreg suggested that adoptive iTreg therapy may not lead to tumor progression in the treatment of autoimmune diseases.

## Discussion

Regulatory T cells are master immunoregulatory cells involved in the maintenance of tumor microenvironments. Their role in maintenance of immune tolerance and tumor evasion has been well-established in a variety of studies (55). However, research has been limited by the scarcity of cells. Here, we report a rapamycin-based protocol to produce *in vitro* Treg cells from colorectal cancer patients and single-cell transcriptome/TCR sequencing results of Treg cells. Rapamycin is an inhibitor of the mammalian target of rapamycin complex 1 (mTORC1) and is clinically used to prevent GVHD in transplantation and autoimmune disease treatment (3, 56). The mechanistic target of rapamycin is a conserved intracellular serine/threonine kinase belonging to the phosphoinositide 3-kinase- (PI3K-) related kinase family. Recent studies have shown that rapamycin can inhibit the proliferation of Teff including Th1 and Th17 cells, support the proliferation of Treg, and enhance the function of FoxP3+ Tregs (3, 57). Thus, rapamycin has been used in clinical studies to amplify Treg cells *in vitro* for the treatment of autoimmune diseases. In our study, initial CD25+ cells were expanded in the presence of anti-CD3/anti-CD28 Dynabeads, IL-2 and rapamycin, reaching a median of 75 (range, 20–105) fold expansion with CD4+CD25+CD127^dim/−^ cells exceeding 90.0%. CD4+CD25+FoxP3+ accounted for >60% of the amplified Treg cells. CFSE-labeled coincubation function experiments showed that expanded iTregs exhibited strong *in vitro* suppression of CD8+T cell proliferation.

However, despite the strong immunosuppressive effect of *in vitro* expanded iTregs, it is unclear whether iTregs are similar to pTregs in terms of gene expression and TCR diversity or whether iTregs are derived from proliferative pTregs. Our single-cell transcriptome data, together with the complete TCR information for over 3,000 cells in each sample, provided a comprehensive resource for exploring the multidimensional properties of Treg cells. Integrated transcriptome analysis showed that the RNA expression profiles of pTreg and iTreg cells were interlaced, and functional enrichment analysis of differentially expressed genes demonstrated that pTregs exhibited enhanced suppressive function and iTregs exhibited an increased proliferation ability. Pseudo-time trajectory analysis revealed that pTregs are mainly divided into three states: activated/effector, resting, and proliferative Tregs. In contrast, expanded iTregs are a mixture of proliferating and activated/effector Tregs. This may be due to differences of the cell microenvironment *in vivo* and *in vitro*. Although activated/effector Tregs displayed increased percentages of cells that expressed “Treg signature genes” at a higher level, such as CTLA-4, FoxP3, and TNFRSF9 (19), we think the possibility that this phenotypic analysis does not truly distinguish between the two different subsets but just a difference in Treg cell differentiation status. Thus, it remains possible that resting Tregs may represent a population of Treg precursor that developed into fully functioning Tregs upon activation. The *in vitro* expansion of iTregs is a dynamic and continuous process. Under the action of IL-2 and rapamycin, the cells proliferated and gradually developed into effector Treg cells. Consistent with our findings, a recent single-cell expression analysis of human and mouse Tregs also suggested that Treg cells form a continuum gravitating around three major poles in their research and noted that one axis encompasses the resting/activated difference reflected by CCR7 or CD62L (58). A single RNA-Seq and TCR tracking analysis conducted by Professor Zhang's team found that tumor-infiltrating Tregs were among the highly expanded populations in colorectal cancer. Most clonal colorectal cancer infiltrating Tregs (88%) harbored TCR clonotypes exclusive to themselves, which indicated their potential for recognizing tumor-associated antigens and local expansion characteristics (59). Kamada et al. also found that proliferative (MKi67+) Treg cells may promote tumor hyperprogressive disease (HPD) during anti-PD-1 immunotherapy and indicated that the inhibition of Treg cell proliferation could be an important strategy to treat and prevent HPD (60).

A direct approach to determine the relationship between iTregs and pTregs involves a comparison of their TCR repertoires (41). TCR repertoire analysis indicated minimal overlap between iTreg and pTreg cells (*P* = 0.0001), and only three shared TCR clones were noted in our single-cell sequencing data. TCR repertoire comparisons indicated that pTregs were not the source of iTreg cells, supporting the notion that *in vitro* expanded iTregs were mainly transformed from activated CD4+CD25+ cells under the action of rapamycin, IL-2 and anti-CD3/anti-CD28 expander beads in the culture system. However, as TCR repertoires can add only partial information as for the originality of iTregs, e.g., pTreg vs. inducible CD4+CD25+ cells. We used additional methods to support our hypothesis that expanded iTreg cells were mainly transformed from CD4+CD25+ T cells rather than pTreg proliferation. We amplified pure pTreg cells according to the iTregs expansion protocol, but no obvious CFSE-labled proliferation was observed. CFSE-labeled proliferation of iTregs and pTregs without the addition of rapamycin, IL-2, and anti-CD3/anti-CD28 expander beads in the medium also showed no proliferation. Our hypothesis was also supported by an *in vitro* expansion study of Treg cells for the treatment of autoimmune and inflammatory diseases (13, 61). In their study, they stained day 0 enriched CD25+ cells and iTreg cells at day 21 with a panel of 24 distinct TCR Vβ monoclonal antibodies, representing ~70% of the human TCR Vβ repertoire. And found that both CD25+ cells at day 0 and iTregs at day 21 expressed all the 24 TCRs (30). In addition, Motwani et al. demonstrated that effector Tregs could clonally expand in the late decidua during normal pregnancy, but could not expand in peripheral blood of humans (62). Therefore, the expanded iTreg cells were mainly transformed from CD4+CD25+ T cells rather than pTreg proliferation.

Differences in chemokine receptor expression in Treg cells have also been observed. Multiple chemokine receptors were highly upregulated in pTreg cells, indicating that the compartmentalization and trafficking of natural Treg cell may exhibit tissue or/and organ specificity (24). Increasing evidence indicates that specific depletion or functional modulation of Tregs evokes effective tumor immunity (63, 64). Intra-tumor injection of anti-CCR10 immunotoxin blocks the CCL28 and CCR10 interaction, reduces the accumulation of Treg cells in TME, and enhances the anti-tumor immune response in the mouse model (22). Seminal studies have confirmed that the chemokine receptors, such as CCR4, function non-redundantly to enable the transport of Treg to non-lymphoid tissues, such as lung and skin, and that this function is essential for maintaining immune homeostasis specifically at these sites (27). Cell-depleting anti-CCR4 mAb therapy specifically depletes CCR4+Tregs and induces the expansion of CD8+ T cells that respond to tumor-associated antigens *in vivo* and *in vitro* (65). Similarly, treatment with anti-CCR8 mAb significantly suppressed tumor growth and improves long-term survival in colorectal tumor mouse models (66).

Previous studies demonstrated that CCL28 secreted by hypoxic tumor cells recruited Treg cells to the neoplastic lesion by interacting with CCR10 in ovarian and liver cancer (22, 23). Several studies have also confirmed that CCL28 was overexpressed in colorectal cancer tissues and associated with poor prognosis (52–54). Our study found that effector pTregs characteristically expressed the chemokine receptor CCR10, which was not expressed in iTregs. We used flow cytometry analysis to confirm that CCR10+pTreg ratio was higher in peripheral blood of colorectal cancers than that in other cancer types. Multiplex fluorescent IHC confirmed that CCL28 was significantly correlated with intratumoral CCR10+Treg infiltration, and high CCL28 expression and intratumoral CCR10+Treg infiltration were predictors of short PFS. However, FOXP3 was not a prognostic factor for PFS. The role of Treg cells was controversial in colorectal cancer patients, and Foxp3 (+) T cell infiltration has suggested a better prognosis in some studies. Saito et al. categoried tumor-infiltrating Treg cells into two categories in colorectal cancer: FoxP3^low^ and FoxP3^hi^, and patients with abundant infiltration of FoxP3^low^ T cells in colorectal cancer had significantly better prognosis than those with predominantly FoxP3^hi^ Treg cell infiltration (16). Our study identified a more specific Treg cell phenotype (CCR10+Treg cells) to predict survival in patients with colorectal cancers. CCR10+ was up-regulated in activated/effector pTregs and interacted with tumor-secreted CCL28 to mediate the migration of Treg cells to neoplasm and promote tumor progression. Therefore, depletion of CCR10+ effector Treg cells from TME could produce beneficial anti-tumor immunity, which may be a promising strategy for the treatment of colorectal cancer. What's more, the relative lower trafficking receptor expression and loss of CCR10 expression in expanded iTregs suggested that adoptive iTreg therapy may not lead to tumor progression in the treatment of autoimmune diseases. Clinical trials of adoptive transfer of iTreg cells to prevent GVHD demonstrated that iTreg cells did not hamper the anti-tumor activity of conventional CD4+ and CD8+ T cells while preventing GVHD compared with control patients (9, 67). We asked whether adoptive iTreg therapy could be used to prevent and treat life-threatening autoimmune related adverse events (irAEs) when treated with PD-1/PD-L1 or CTLA-4 immuno-checkpoint inhibitors. Currently, engineered *in vitro* expanded iTregs with overexpression of organ or tissue specific homing receptors robustly suppressed ongoing experimental autoimmune diseases (68). The generation of gut-Homing Treg cells overexpressing CCR9 exhibited strong inhibitory ability in mouse colitis, representing a promising novel therapy for autoimmune diseases (69). Our data distinguished the transcriptomic characteristics of different groups of Treg cells and revealed the context-dependent functions of different subsets of Treg cells. This information is critical to the development of alternative therapeutic operational strategies for Treg cells in autoimmune disease and cancer (11).

## Conclusion

In our study, we demonstrated successful *in vitro* expansion of iTreg cells in the presence of anti-CD3/anti-CD28 Dynabeads, IL-2, and rapamycin from PBMCs of patients diagnosed with colorectal cancers. Phenotypic identification of the harvested cells met the iTreg evaluation criteria, and functional assays showed that the expanded iTregs significantly inhibited the proliferation of CD8+T cells *in vitro*. Single-cell sequencing analysis demonstrated that the transcriptome of pTreg and iTreg cells were interlaced. Specifically, pTregs were more suppressive, whereas iTregs were more proliferative. Pseudo-time trajectory analysis revealed that pTregs were mainly divided into three branches: activated/effector, resting and proliferative Tregs. In contrast, expanded iTregs included a mixture of proliferating and activated Tregs. TCR repertoire comparison revealed minimal overlap between iTreg and pTreg cells. Multiple chemokine receptors were highly upregulated in pTreg cells. CCR10 was overexpressed in activated/effector pTregs. Multiplex fluorescent IHC confirmed that CCL28 was significantly correlated with intratumoral CCR10+Treg infiltration, high CCL28 expression and intratumoral CCR10+Treg infiltration were poor predictors of PFS. CCR10 guided pTreg migration to tumors via interactions with tumor secreted chemokine CCL28 to promote tumor progression. Depletion of CCR10+Treg cells from TME could be used as an effective treatment strategy for colorectal cancer patients. The relatively reduced expression of iTreg cells and loss of CCR10 expression in expanded iTregs suggested that iTregs exhibit poor organ specificity, and iTreg infusion may not lead to tumor progression in the treatment of autoimmune diseases. Current total Treg expansion or Treg targeting therapy with limited specificity have demonstrated their potential for autoimmune and cancer therapy (11). Our study compared the single-cell transcriptome characteristics of pTregs and iTregs, this information will help to improve strategies targeting specific Treg subsets without adversely affecting other effector cells or Treg cells, paving the way for Treg depletion, and Treg modification therapy.

## Data Availability Statement

The raw data supporting the conclusions of this article will be made available by the authors, without undue reservation.

## Ethics Statement

The studies involving human participants were reviewed and approved by Ethics Committee of Tianjin Medical University Cancer Institute and Hospital. The patients/participants provided their written informed consent to participate in this study.

## Author Contributions

ZH and JZ performed cell culture, single-cell analysis, and wrote paper. YZ and LY supported data interpretation. YA isolated single cells. WY performed flow cytometry analysis. FW and XR conceived and guided the project. All authors contributed to the article and approved the submitted version.

## Conflict of Interest

The authors declare that the research was conducted in the absence of any commercial or financial relationships that could be construed as a potential conflict of interest.
